# Predictors of higher pain in possible open globe injury emergency medical services activations

**DOI:** 10.1007/s10792-025-03418-4

**Published:** 2025-01-31

**Authors:** Hassaam S. Choudhry, David Mothy, Aneesh Reddy, Aman M. Patel, Skyler Peterson, Benjamin Fisher, Mohammad H. Dastjerdi

**Affiliations:** 1 Department of Ophthalmology and Visual Science, Rutgers New Jersey Medical School, Newark, NJ USA; 2 The College of New Jersey, Ewing, NJ USA; 3 Department of Pediatrics, National Emergency Medical Services (NEMSIS) Technical Assistance Center, The University of Utah School of Medicine, Salt Lake, UT USA

**Keywords:** Ocular trauma, Open globe injury, NEMSIS, Pain, Race, Gender

## Abstract

**Purpose:**

To determine predictors of high pain in open globe injury (OGI) cases treated and transported by Emergency Medical Services.

**Methods:**

The National Emergency Medical Services Information System database was queried for activations of OGI between 2017 and 2021. Demographic, location, medication, and date and time information was collected. Cases were divided based on the intensity of maximum pain reported (low pain: below 5/10, high pain: above 5/10). Logistic regression was used to identify significant predictors of high maximum pain.

**Results:**

Of 53,589 cases of OGI, 20,766 reported high levels of pain. Females were more likely to report high pain than males (OR 1.24, CI 1.195–1.285). All age groups between 16 and 75 years old were more likely to report high pain than patients below 15, while all age groups above 75 were less likely. American Indians/Alaska Natives, Black, and Hispanic Americans were all more likely to report high pain than White Americans (American Indian, OR 1.249, CI 1.067–1.461; Black, OR 1.332, CI 1.277–1.390; Hispanic, OR 1.133, CI 1.064–1.207). OGI cases in the Midwest and South regions were less likely to report high pain than those in the West (Midwest, OR 0.868, CI 0.807–0.933; South, OR 0.800, CI 0.748–0.855). Compared to low pain patients, a greater percentage of high pain patients received opioid analgesia (10.04% vs. 0.44%).

**Conclusions:**

Demographic factors and location may contribute to higher pain in OGI patients. This information may prove useful in the management of OGI and may warrant further investigation into the nature of open globe trauma.Kindly check and confirm the inserted city is correct for affiliation 3.Correct

## Introduction

Open globe injury is a major cause of visual complications in the United States. Hospitalizations due to eye injury have increased significantly in past years [1] and recent epidemiological studies now estimate that up to 7.5% of Americans will experience an eye injury at some point in their lives [2, 3]. The clinical outcomes of open globe injury have been well documented in the hospital and out-patient settings. Patients with ocular injury are at high risk for complications that reduce quality of life and visual acuity in the long-term [4], though the severity of visual impairment is heavily associated with the nature and severity of the initial insult [5]. At the same time, little attention has been given to studying the nature of pain in ocular trauma.

Pain is a common symptom reported in cases of open globe injury and has been demonstrated to be an indicator of severity in certain settings of ocular trauma [6, 7]. One recent study showed that 90% of cases of eye trauma presenting to the Emergency Department presented with eye pain as the chief complaint [8]. Given that pain is a common symptom of ocular trauma and can cause patients significant distress, it is worth examining specific factors that may predict increased severity of reported pain in ocular trauma injuries such as open globe injuries (OGIs). In this study, we seek to characterize predictors of pain in OGI cases reported to emergency medical services (EMS) available in the National Emergency Medical Services Information System (NEMSIS) database.

## Methods

NEMSIS is a federally funded program created to standardize EMS patient care reporting via a state and national database to assess and improve EMS systems [9]. The NEMSIS database was queried for all EMS activations of isolated open globe injury between the years 2017 and 2021. Demographic information for each of these cases was extracted and cases were divided based on the intensity of maximum reported pain. Pain scores were reported on a 10-point scale, with 0/10 being no pain at all and 10/10 being the worst pain ever experienced. A maximum self-reported pain score higher than 5/10 was classified as high pain, and a score lower than 5/10 was classified as low pain. Logistic regression was used to determine statistically significant indicators of high pain. The medications administered in each case were also recorded and classified into one of five categories: opioids, non-opioid analgesics, antiemetics, sedatives, and other. Cases consisting of cancellations, assists, and standby cases were excluded.

## Results

Query of the NEMSIS database returned 53,589 cases of OGI reported between 2017 and 2021. 54% of these cases were males and 46% were females. 59% of these cases were White Americans, 28% were Black or African Americans, and 9% were Hispanic or Latino, with less than 3% of cases accounting for individuals of all other races. OGI cases were recorded across all age groups, with a slight plurality occurring in the 26 to 35 age group (18%). A plurality of cases happened during weekdays (69%) and between the hours of 3:00 pm and 11:00 pm (43%). 84% of cases were classified as emergent, requiring an immediate response. EMS activation emergent status is a triage metric determined by EMS dispatch services to indicate the relative acuity of an emergency Most cases were reported in the Southern United States (63%) and in urban areas (82%). The median maximum pain experienced was 4.0/10 (See Table 1).

**Table 1:** Characteristics of patients with open globe injuries treated and transported by EMS, 2017–2021

Variables	Overall (N = 53,589)
Categorical age	
1–15	3792 (7%)
16–25	8028 (15%)
26–35	9439 (18%)
36–45	7191 (13%)
46–55	5962 (11%)
56–65	5963 (11%)
66–75	4712 (9%)
76–85	4781 (9%)
> 85	3721 (7%)
Sex	
Female	24,665 (46%)
Male	28,924 (54%)
Race	
American Indian or Alaska Native	690 (1%)
Asian	542 (1%)
Black or African American	15,134 (28%)
Hispanic or Latino	5077 (9%)
Multi Race/Ethnicity	194 (0%)
Native Hawaiian or Other Pacific Islander	119 (0%)
White	31,833 (59%)
Day of week	
Weekday	36,845 (69%)
Weekend	16,744 (31%)
Time	
15:00–22:59	22,913 (43%)
23:00–6:59	13,139 (25%)
7:00–14:59	17,537 (33%)
Response mode	
Emergent (Immediate Response)	45,195 (84%)
Emergent Downgraded to Non-Emergent	795 (1%)
Non-Emergent	7450 (14%)
Non-Emergent Upgraded to Emergent	149 (0%)
US census region	
Midwest	11,469 (21%)
Northeast	3983 (7%)
South	33,597 (63%)
West	4540 (8%)
Urbanicity	
Rural	4875 (9%)
Suburban	3700 (7%)
Urban	43,714 (82%)
Wilderness	1300 (2%)
Max Pain Median (q1, q3)	4.0 (1.0, 6.0)

Of 53,589 total cases, 20,766 (39%) reported high maximum levels of pain. Emergent cases were 1.20 times more likely to report high levels of pain than non-emergent cases (CI 1.135–1.260). Females were 1.24 times more likely to report high levels of pain than males (CI 1.195–1.285). All age groups between 16 and 65 years were more likely to report high levels of pain than patients between one and 15 years of age, with the 26 to 35 age group showing the highest likelihood (OR 1.717, CI 1.585–1.859). Patients between the ages of 76 and 85 and patients older than 85 were both less likely to report high levels of pain than patients between the ages of 1 and 15 (76–85, OR 0.699, CI 0.635–0.770; > 85, OR 0.596, CI 0.536–0.661). Patients in rural areas or the wilderness were both more likely to report high levels of pain than patients in suburban areas (rural, OR 1.207, CI 1.104–1.319; wilderness, OR 1.366, CI 1.198–1.557). No significant association was found for urban areas. American Indians/Alaska Natives, Black, and Hispanic Americans were all more likely to report high pain than White Americans (American Indian, OR 1.249, CI 1.067–1.461; Black, OR 1.332, CI 1.277–1.390, Hispanic, OR 1.133, CI 1.064–1.207). No significant associations were found with any other races. Cases in the South and Midwest were less likely to report high pain than those in the West (South, OR 0.800, CI 0.748–0.855; Midwest, OR 0.868, CI 0.807–0.933) (See Table 2).

**Table 2:** Odds ratio estimates for predictors of high pain in open globe injury cases treated and transported by EMS, 2017–2021

Comparison	Odds ratio Point estimate	95% Wald confidence limits	
Gender: Female versus Male	1.239	1.195	1.285
Urbanicity: Rural versus Suburban	1.207	1.104	1.319
Urbanicity: Urban versus Suburban	0.939	0.875	1.008
Urbanicity: Wilderness versus Suburban	1.366	1.198	1.557
Age Category:16–25 versus 1–15	1.450	1.336	1.573
Age Category: 26–35 versus 1–15	1.717	1.585	1.859
Age Category: 36–45 versus 1–15	1.631	1.502	1.772
Age Category: 46–55 versus 1–15	1.577	1.448	1.718
Age Category: 56–65 versus 1–15	1.295	1.188	1.411
Age Category: 66–75 versus 1–15	0.926	0.844	1.016
Age Category: 76–85 versus 1–15	0.699	0.635	0.770
Age Category: > 85 versus 1–15	0.596	0.536	0.661
Response Mode: Emergent (Immediate Response) versus Non-Emergent	1.196	1.135	1.260
Response Mode: Emergent Downgraded to Non-Emergent versus Non-Emergent	0.859	0.734	1.007
Response Mode: Non-Emergent Upgraded to Emergent versus Non-Emergent	1.097	0.781	1.542
Race: American Indian or Alaska Native versus White	1.249	1.067	1.461
Race: Asian versus White	0.910	0.759	1.091
Race: Black or African American versus White	1.332	1.277	1.390
Race: Hispanic or Latino versus White	1.133	1.064	1.207
Race: Multi Race/Ethnicity versus White	1.322	0.991	1.764
Race: Native Hawaiian or Other Pacific Islander versus White	0.933	0.637	1.365
US Census Region: Midwest versus West	0.868	0.807	0.933
US Census Region: Northeast versus West	1.025	0.937	1.122
US Census Region: South versus West	0.800	0.748	0.855
Time: 15:00–22:59 versus 7:00–14:59	0.999	0.959	1.042
Time: 23:00–6:59 versus 7:00–14:59	0.984	0.938	1.032
Day Of Week: Weekday versus Weekend	1.009	0.971	1.049

A majority of cases did not specify the administration of any medications (74% for high pain, 90% for low pain). When compared to low pain cases, a greater percentage of high pain cases specified the administration of opioids (10.04% vs. 0.44%), non-opioid analgesics (1.44% vs.0.46%), anti-emetics (3.91% vs. 1.05%), and sedatives (1.00% vs. 0.60%) (See Table 3).

**Table 3:** Medications administered in open globe injury cases treated and transported by EMS, 2017–2021

Drug class	High pain	Low pain
None/Not specified	17,227 (72.92%)	30,545 (90.44%)
Opioid	2373 (10.04%)	148 (0.44%)
Non-opioid analgesic	341 (1.44%)	157 (0.47%)
Antiemetic	923 (3.91%)	354(1.05%)
Sedative	237 (1.00%)	201 (0.60%)
Other	2517 (10.65%)	2363 (7.00%)

## Discussion

A slight majority (54%) of OGI cases occurred in men, which is consistent with the current literature on the epidemiology of eye injury in the United States [10]. A slight majority of OGI cases occurred in white Americans (59%), followed by Black and African Americans (28%), which is consistent with one past study examining trends in eye injury through analysis of National Center for Health Statistics (NCHS) surveys [11]. The racial distribution of OGI cases has not been otherwise characterized in the literature. A plurality (46%) of cases occurred in individuals between the ages of 16 to 46, which is consistent with the literature’s understanding that eye injuries largely occur in young adults [12]. OGI’s were reported in disproportionate amounts in the South (63% of injuries from 39% of the population [13]), which may be due to its warmer weather which has been shown to be associated with increased incidence of ocular injury [14]. Though a large majority of cases happened in urban environments, this is proportional to the population distribution of the United States (83% of injuries from 80% of the population). The average maximum pain reported in these cases was 4.0/10, which is below the national average of 6.49/10 for all pain-related visits to the emergency department [15]. This could suggest that patients are more likely to seek emergency medical care at lower levels of pain in the context of eye injury.

We found that emergent cases were 1.2 times more likely to report high pain when compared to non-emergent cases. This is consistent with the literature’s understanding that pain can be an indicator of severity in ocular trauma [7]. Additionally, females are more likely to report high pain than men, though the reason for this may be multifactorial. For one, differences in reported level of pain could stem from differences in injury sustained. A recent retrospective study conducted at Massachusetts Eye and Ear Infirmary demonstrated significant gender differences in the etiology of eye injury. For example, men were more likely to have eye injuries that were completely localized to the cornea (46.2% vs. 23.4%), while women were more likely to have eye injuries involving the posterior globe (46.8% vs 28.3%) [10]. Additionally, there are certain etiologies of open globe injury that may be more common in women. A previous study showed that wound dehiscence may occur more frequently in women [16], which is significant given the poor prognosis of such injuries [17]. Such differences could explain the observed difference in reported pain scores between men and women. It is also important to consider how prehospital pain management may influence maximum pain score. A previous analysis of the Wyoming Ambulance Trip Reporting System (WATRS) database found that women were ten percent less likely than men to receive opioid pain medication by prehospital providers in the context of an emergent injury [18]. Though no such study has yet been done in the context of ocular trauma, it can be reasoned that inadequate and unequal pain treatment may explain the greater likelihood of reporting high pain in female cases.

When compared to patients under 16 years of age, all age groups below 66 were more likely to report high pain and all age groups above 75 were less likely to report high pain. Though previous studies have suggested that elderly patients may have higher pain thresholds than younger ones [19], differences in pain sensitivity cannot explain why patients between the ages of 16 and 65 were more likely to report high pain than those below 16. Previous investigations have suggested that children are generally more sensitive to pain than adults [20] This being the case, it is likely that this difference in reported pain scores can be explained by differences in traumatic etiology. Pediatric patients with OGI most often have Zone 1 injuries, involving just the cornea and limbus [21], which may explain their lower likelihood to report high pain.

Patients in rural areas or the wilderness were more likely to report high pain in OGI cases than those in suburban areas. The relationship between regional density and ocular trauma has not been previously investigated, so the reason for this disparity cannot be confidently ascertained. It is possible that this difference in reported pain may be due to the nature of injuries sustained in rural environments. Though no study has characterized differences in ocular trauma between rural and non-rural environments, past studies have shown region-specific differences in generalized trauma. For example, one study has shown that patients in rural settings are more likely to suffer blunt injuries and less likely to suffer penetrating injuries than urban patients [22]. Similar investigation into the nature of ocular trauma in rural environments could help better contextualize why rural patients are more likely to report high pain. Additionally, response time may also play a role. EMS response time in rural environments can be up to double that of suburban and urban environments, which may contribute to higher levels of reported pain to EMS providers [23]. Future investigations should further evaluate the explicit relationship between reported pain scores and both response time and transport time. Patients in the South and Midwest regions were found to be less likely to report high pain in OGI than patients in the West. As with regional density, the reason behind this association remains unclear.

American Indians/Alaska Natives, Black, and Hispanic Americans were all more likely to report high pain than White Americans. As with sex, it is possible that this association may be explained by differences in traumatic etiology. A previous investigation of the National Inpatient Sample (NIS) found that certain races saw higher frequencies of certain types of OGI. For example, 20.5% of OGIs in Native American patients had rupture, while only 15.8% had rupture in white patients. Similarly, 16.6% of OGI cases in Hispanic patients also reported intraocular foreign body, while that was only true of 10.6% of OGIs in white patients [24]. Differences in reported pain by race may also be accredited to inadequate pain management by prehospital providers. Previous studies have found that African American, Hispanic, and American Indian/Native Alaskan patients are all less likely to receive opioid pain medication than White patients in the management and transport of an injury case [18, 25].

High pain cases reported a higher frequency of administration for opioids, non-opioid analgesics, antiemetics and sedatives. This likely reflects higher severity of injury in high pain cases, which may necessitate pain management interventions. Opioid medication was given in 10.04% of high pain cases, which is higher than the percentage given to general acute trauma cases (7.0%) [26].

In discussing pain intensity in the context of OGI, it is necessary to acknowledge its limitations as a diagnostic and prognostic tool. Though pain intensity has been shown to have value as an indicator of ocular injury severity in certain contexts [6], there are situations in which it may prove less useful. For example, a surface level injury to the particularly sensitive cornea may produce more pain than a deeper laceration affecting less innervated parts of the eye, resulting in a seemingly paradoxical relationship between pain intensity and severity of injury [27]. As such, it is important that pain intensity scores in ocular trauma are interpreted with the context of a thorough eye exam.

Limitations of this study are mostly related to the nature of the NEMSIS database. NEMSIS is a “convenience sample” of EMS data from participating agencies that meet the selection criteria. As such, the data from NEMSIS cannot be generalized to the United States population at large [28]. Additionally, differences in reporting protocols between states and EMS agencies may account for selection and information biases, especially when comparing different regions. One of the major limitations of the data is the lack of a clinical diagnosis of OGI by a physician, but as the label of open globe injury is instead made based on the primary impression of the EMS responder. Hence, a proportion of the cases included in the analysis may not specifically be an OGI but some other type of eye injury. Nevertheless, the conclusions we draw from these results are generalizable to all of ocular trauma. Finally, our study is limited by the variables available for analysis in NEMSIS which restricts our ability to perform more epidemiological or clinical outcomes focused research. Such limitations are present in many of the articles published utilizing the NEMSIS database and are the consequences of using such big data registries. Limitations in the data available may restrict some of the conclusions that can be made as seen, however the conclusions we are able to make are well supported by the breadth of sampling agencies used by NEMSIS, as well as the database’s thorough selection criteria.Kindly check and confirm the Author contributions, Conflict of interest, Funidng is correctly identified.correct

In all, this study highlights significant relationships between maximum self-reported pain in OGI, and a wide breadth of demographic, geographic, and circumstantial factors. Future investigations may seek to focus on fewer co-variables, thereby outlining a specific patient profile at risk for severe pain. Such an analysis would build on these findings to provide actionable information to emergency medical physicians and ophthalmologists.

## Data Availability

No datasets were generated or analysed during the current study.
